# Morphometric delineation of administrative boundaries and classification of threatened categories of small watersheds in transboundary rivers

**DOI:** 10.1038/s41598-023-28913-5

**Published:** 2023-01-30

**Authors:** Anshumali, Rahul Kumar Pandey, Rahul Kumar Gupta, Sanchit Kumar

**Affiliations:** Laboratory of Biogeochemistry, Department of Environmental Science and Engineering, Indian Institute of Technology (Indian School of Mines), Dhanbad, Jharkhand 826004 India

**Keywords:** Climate sciences, Environmental sciences, Hydrology

## Abstract

The ecological conservation of large rivers is impossible unless immediate attention is given to protecting their small tributaries at local levels. The natural boundaries of large river basins are shrinking because their tributaries and streams of different orders are disappearing at an unprecedented rate. Delineation of the fixed administrative boundaries (AB) to protect the natural boundary of small rivers and their classification into appropriate threatened categories, the present study was carried out on the 54.08 km long Banki River in the Ganga River basin. The > 70% irreversible loss in the number of streams (Nu), length of streams (Lu), and drainage density (Dd) resulted in the conversion of the 6th order Banki into the 4th order river. The extreme morphometric changes result in the Banki watershed being under the “Critically Endangered” category. The drainage density ratio (DdR) and mean stream width (M_sw_) were used to determine the width of AB (W_AB_). The “River Red List Categories and Criteria” are being proposed to strengthen global initiatives at the local levels to protect and conserve inland water bodies and transboundary rivers.

## Introduction

The diversion of small watersheds in transboundary river basins for livelihood is causing alterations in biogeochemical cycles, frequent climate change episodes, loss of biodiversity, decreasing terrestrial and aquatic productivity, and depleting per capita water availability at local, regional, and global scales^1^. Approximately 40% of the global population live in 276 transboundary lake and river basins shared between two or more countries that cover almost one-half of the globe’s land surface and 60% of global water flow^2^. Transboundary rivers create hydrological, social, and economic interdependencies between societies, complicating transboundary water management^3^. In addition, dams and reservoirs and their up and downstream propagation of fragmentation and flow regulation are the leading contributors to the loss of river connectivity in the large river systems (LRSs)^4^ and free-flowing rivers (FFRs)^5^. Such complexities are further aggravating due to the disappearing small watersheds in the transboundary basins irrespective of physical versus social water scarcity, upstream versus downstream, and water stress versus water shortage^6^. Further, transboundary rivers on the local, regional, and global scales are considered a natural means of aquifer recharge^7^, where over 70% of India’s food grain production is groundwater-dependent^8^ and exceeds groundwater abstraction from the USA and China^9^. As a result, the underpinning future well-being of transboundary rivers is embedded in the protection and conservation of their small rivers’ morphometric patterns to sustain an ecological trade-off between the water-energy-food nexus^10^.

The first and foremost question is how to protect the natural boundaries of small rivers or watersheds. The delineation of fixed administrative boundary (AB) is critical to protecting the natural boundaries of streams of different orders forming the small, medium, or large watersheds^11^. A few research attempts have been made to delineate the river corridors protecting the morphology of the main channel^12,13^. However, the intrinsic morphometric parameters have never been used to define the AB for every stream order, i.e., a holistic approach is a prerequisite to quantifying the width of AB. The AB is important from an implementation point of view^14^ because it offers a piece of land statutorily to protect the morphometric diversity of any watershed within and outside the protected areas^15^.

The second most important question is how morphometric parameters can help conserve the existing morphometry from further extinction under changing land use and land cover (LULC) scenarios. The comparative assessment of morphometry of the reference year (1977) with current morphometry (2021), especially the linear aspects and drainage texture, can help prepare the threatened category and criteria for rivers, tributaries, and streams of a different order, length, and width.

The research questions were tested at the Banki watershed in the lower Vindhyan region of the Ganga River basin. This watershed's linear aspects and drainage texture were analyzed and compared to understand the impact of LULC change on morphometry and river-aquifer interaction. The “drainage density ratio” (DdR) and “River Red List Categories and Criteria” (RRLCC) were derived from morphometric parameters to delineate the width of AB and define the threatened categories of streams and tributaries, respectively.

## Materials and methods

### Morphometric analysis

Banki river watershed (24° 23′ 6.42″ and 24° 10′ 17.71″ North Latitude and 83° 23′ 9.55″ and 83° 43′ 19.96″ East Longitude) is an integral component (518.35 km^2^) of the downstream Ganga River basin at a distance of ≈ 198.44 km southeast in the Garhwa district of Jharkhand, India. The 54.08 km long Banki River originates from the Sagma hills (southwest) and the confluence with the North Koel (northeast). The altitude varied from 129 to 493 m above mean sea level in the watershed (Supplementary Fig. S1).

The five Survey of India (SOI) toposheets (63P/7, 63P/8, 63P/11, 63P/12, and 63P/15; Scale—1:50,000; 1977) were downloaded (https://onlinemaps.surveyofindia.gov.in/)^16^ and georeferenced based on Universal Transverse Mercator (UTM) projection and the World Geodetic System (WGS) 1984 UTM Zone 44N datum. All georectified toposheets were mosaicked using Arc GIS 10.2 software. For the drainage network extraction, the mosaic toposheets were manually digitized using Arc GIS 10.2 editing tool. Further, the digitized shapefile was converted into a topology for the error correction^17^ and then filled attributes for each stream based on the Strahler method of stream ordering^18^. The un-branched streams were designated as 1st order streams; two 1st order streams joined to form 2nd order streams, the joining of two 2nd order streams resulted in a 3rd order stream, and so on. The watershed and sub-watershed delineation were carried out with the help of pour points in the Arc Hydro tools using ASTER (Advanced Spaceborne Thermal Emission and Reflection and Radiometer) 30 m spatial resolution digital elevation data set (downloaded from https://search.earthdata.nasa.gov/search)^19^ and cross-checked with toposheet contours.

The drainage density, drainage source, drainage confluence, and drainage frequency maps were prepared with the manual interpretation of total stream length^18^, total 1st order streams^18^, total confluence streams, and the total number of streams^20^, respectively, in a 1 km × 1 km fishnet grid and put them into the point shapefile. The inverse distance weighting (IDW) interpolation^21^ was used to create the final maps for the reference year (1977) in Arc GIS 10.2 software.

The field inventory updated the current morphometric details using a global positioning system (GPS, Model Garmin eTrex 30) and Drone surveying (DJI AIR 2S) in November–December 2021. The qualitative and quantitative verifications involved the presence/absence of streams along with their origin and confluence points. The recorded field data were used to delete or retain streams in digitized shapefile and depict changes in the drainage network. Associated spatial data were generated by repeating the exercises of GIS processing using Arc GIS 10.2 software.

The drainage network data of the reference year (1977) and current year (2021) were compared to illustrate the changes in linear aspects (stream order, number of streams, length of the streams, bifurcation ratio, and Rho coefficient)^18,20^ and drainage texture parameters (drainage density, stream frequency, drainage texture, constant of channel maintenance, and infiltration number)^20,22,23^ of the Banki watershed and sub-watersheds. The ASTER DEM data was used in the calculation of elevation and perimeter^22^.

### GWPZ mapping

To understand interactions between river-aquifer, the groundwater potential zone (GWPZ)^24^ was delineated where ten input variables were used under two LULC scenarios (1991 and 2021), two rainfall (1961–1990 and 1991–2020), and two drainage densities (1977 and 2021) patterns: the slope was generated from ASTER DEM data (pixel size = 30 m resolution); the shapefiles of geomorphology, geology, lithology, and lineament density were taken from the Bhukosh portal (https://bhukosh.gsi.gov.in/Bhukosh/MapViewer.aspx)^25^; drainage density from SOI toposheets^16^ and field data; soil texture was collected from FAO soils portal (https://storage.googleapis.com/fao-maps-catalog-data/uuid/446ed430-8383-11db-b9b2-000d939bc5d8/resources/DSMW.zip)^26^; average annual rainfall maps (30 years: 1961–190 and 1991–2020) were prepared by collecting rainfall data (0.25° × 0.25° latitude–longitude resolution) from India Meteorological Department (IMD) monitored rain gauge stations^27^. The LULC maps were prepared by unsupervised classification^28^ of 7 bands of the Landsat 5 satellite dataset (26 Sep 1991)^29^ and 11 bands of the Landsat 8 satellite dataset (14 Oct 2021)^30^ using iterative self-organizing data analysis technique algorithm (ISODATA)^31^ performed with 200 spectral classes, a convergence threshold of 0.950, and 10 iterations. The Euclidean distance in the feature space assigned every pixel to a cluster through some iterations, which introduces considerable subjectivity into the classification process^32^. The LULC classes include agriculture (lowland and highland crop fields with and without crops); barren (dry and bare with very few plants and no trees); built-up (high, medium, and low-density settlements, dispersed settlements, infrastructures such as schools, hospitals, industries, bridges, and roads); vegetation (forest cover, trees outside forests, road plantation, shrubs, and herbaceous layer); and water (wet and dry rivers, river banks, waterlogged areas, and small ponds).

The artificial neural network (ANN) processing was implemented using the Neural Network ToolBox for MATLAB^33^. The Feedforward neural network structure^34^ selected in this study consists of an input layer (ten input variables described above), a hidden layer (hidden neurons), and an output layer (well water level) for the delineation of groundwater potential zones. The input and target data were introduced into the MATLAB R2020b software, and all raster-format groundwater-related factors and well water levels were converted into ASCII-format files in GIS^35^. Before running the ANN model, we selected the training, testing, and validation data corresponding to 70%, 15%, and 15% of the total study area (575,944 pixels). Six numerical matrices were generated using specific scripts: the X-train-input, Y-train-target, X-test-input, Y-test-target, X-validation-input, and Y-validation-target matrices^36^.

The input data matrices were normalized to train the neural network. The initial weights were randomly selected, followed by the Levenberg–Marquardt back-propagation algorithm^37,38^ to minimize errors between the predicted (target) and calculated output values. The number of epochs was set to 1000, and the mean square error (MSE) of 0.001 was used as the stopping criterion^39^. After multiple tests, the network was optimized to have ten nodes in the input layer, three hundred twenty-five nodes in the hidden layer, and one node in the output layer structure (10 × 325 × 1) at 587 epochs (1991) and 302 epochs (2021). The results showed MSE and correlation (R) 0.005 and 0.73, respectively, for 1991 and 0.006 and 0.71, respectively, for the year 2021. All ten thematic maps were integrated with the weighted overlay analysis method in the GIS platform using the Eq. (1)^40,41^ to generate the GWPZ:

1 $$ {\text{GWPZ }} = \, \sum ({\text{Wi}} \times {\text{Xi}}), $$

where, Wi represents the weight of the thematic layers and Xi represents the rank of the thematic map's subclass.

### Delineation of AB and derivation of RRLCC

Administrative boundaries are highly relevant from an implementation point of view since they capture the hierarchy implicit in authority structures that shape multilevel governance of environmental resources^10,12,13,42^. In this reference, we developed an empirical method for delineating the AB along the left and right bank of streams, irrespective of their order, number, length, and width. After performing all permutations and combinations, we found that the Dd emerged as the most suitable morphometric parameter for the derivation of the AB because Dd is the only parameter that primarily portrays one dimension (Lu) and two dimensions (area, A) of the watershed. The DdR (ratio of Dd_1977_ in the reference year to Dd_2021_ in the current year) was computed to incorporate the unitless "watershed factor" and overcome biases in the estimation of the AB (Eq. 2).

2 $$ {\text{Drainage}}\,{\text{density}}\,{\text{ratio }}\left( {{\text{DdR}}} \right) \, = {\text{ Dd}}_{{{1977}}} /{\text{ Dd}}_{{{2}0{21}}} $$

The width of the administrative boundary (W_AB_) was the product of DdR and mean stream width (M_SW_) (Eq. 3). The M_SW_ was determined by taking into account the width of each stream in 1st, 2nd, and 3rd order at three locations (origin point, midstream, and before confluence point). The M_SW_ of the 4th order main trunk (Banki River) was determined by measuring width at nine locations considering spatial variation in LULC, geomorphology, geology, and soil types in the 1 km × 1 km grided watershed. Finally, the W_RAB_ (width of administrative boundary on the right bank) and W_LAB_ (width of administrative boundary on the left bank) were computed as half of the W_AB_ (Eq. 4).

3 $$ {\text{W}}_{{{\text{AB}}}} = {\text{ M}}_{{{\text{SW}}}} \times {\text{ DdR,}} $$

4 $$ {\text{W}}_{{{\text{RAB}}}} \,{\text{or}}\,{\text{W}}_{{{\text{LAB}}}} = {\text{ W}}_{{{\text{AB}}}} /{ 2}{\text{.}} $$

The essential information to develop the RRLCC, we rigorously reviewed the evolution of the IUCN Red List, where qualitative and quantitative data on the population and habitat of flora and fauna are taken into consideration to define threatened categories and criteria^43^. We also reviewed the six IUCN protected areas management categories^15^ to determine and incorporate the RRLCC in this framework. The proposed concept of the RRLCC deals with the percent change in the Nu, Lu, and Dd in watersheds and sub-watersheds and firmly advocates the protection and conservation of abiotic components like rivers at par with flora and fauna within and outside the protected areas.

## Results and discussion

### Impacts of LULC on river morphometry

To study the factors affecting the morphometry of the Ganga River basin, we selected the small Banki River (length: 54.08 km and watershed area = 518.35 km^2^) in the lower Vindhyan region (24° 23′ 6.42″ and 24° 10′ 17.71″ North Latitude and 83° 23′ 9.55″ and 83° 43′ 19.96″ East Longitude) of India (Supplementary Fig. S1). This river originates from the Chhotanagpur Gneiss Complex^25^, characterized by moderately dissected denudational hills and valleys with a maximum elevation of 493 m, which confluences with the North Koel River at 169 m mean sea level (MSL) towards the northeast direction. The granite gneiss and hard compact clay with caliche nodules^44^ contribute to the formation and deposition of riverbed sediments. High resolution (0.25° × 0.25°) gridded rainfall dataset of the India Meteorological Department (IMD) showed a decrease in annual average precipitation from 1074.63 mm (1961–1990) to 963.73 mm (1991–2020) (Supplementary Fig. S2). The land use and land cover (LULC) change between 1991 and 2021 showed the gradual or random diversion of forested hilly pediplains and streams to agricultural and built-up areas (Supplementary Fig. S3). The vegetation, water bodies, and barren land showed a decline of 13.9%, 3.6%, and 1.6%, respectively, while agriculture land and built-up area increased by 16.8% and 2.4%, respectively (Table 1). The agricultural lands are rainfed and irrigated and covered with a large canopy of scattered tropical deciduous trees. The rural infrastructure development is transforming villages into peri-urban centers. These factors strongly affect the regeneration of forests and rivers, which are tightly linked to rainfall patterns during the monsoon season (June–September).

**Table 1:** Land use and land cover change analysis between 1991 and 2021.

1991				2021				
S no.	Class name	Area (km^2^)	Area (%)	Class name	Area (km^2^)	Area (%)	(%) Change	
1	Agriculture	126.32	24.4	Agriculture	213.26	41.1	16.8	19.2
2	Built up	3.84	0.8	Built up	16.43	3.2	2.4	
3	Barren land	40.73	7.9	Barren land	32.28	6.2	− 1.6	− 19.2
4	Vegetation	311.62	60.1	Vegetation	239.39	46.2	− 13.9	
5	Water	35.83	6.9	Water	17	3.3	− 3.6	
	Total	518.35	100		518.35	100	0	0

**Figure 1:**
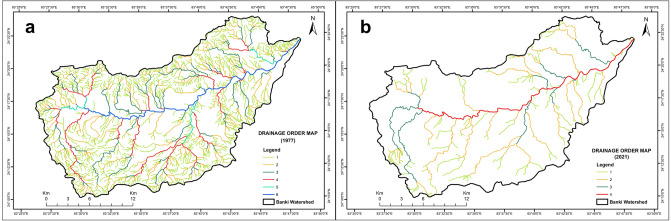
Drainage order map. (**a**) Reference drainage order map extracted from Survey of India toposheet (1977). (**b**) Delineation of drainage order map after ground truth verification of existing streams in 1 km × 1 km grided Banki watershed (2021).

The summary of linear morphometric parameters is given in Table 2. The number of streams (Nu)^20^ decreased from 1511 (1977) to 175 (2021), while the total length of streams (Lu)^18^ shrank from 1286.92 km (1977) to 381.95 km (2021), resulting in the conversion of the 6th order Banki into the 4th order river (Fig. 1). The 1st order streams still exist on the low dissected structural hills without forming 2nd order streams. Moreover, the heterogeneous dendritic drainage network is transformed into flat and homogenized terrain dominated by agriculture practices and rural and periurban settlements. Now, the 2nd and 3rd order streams become 1st order streams, and 3rd and 4th order streams are enumerated as the 2nd order streams, and so on. This trend is disrupting the continuum of morphological and hydrological features from the headwaters to the mouth^11^ and the consequent unpredictable impact on water level pulsing on the resulting floodplain, i.e., “aquatic/terrestrial transition zone” (ATTZ)^45^ in the Ganga River Basin. The increase in the mean bifurcation ratio (Rbm)^18^ from 4.14 (1977) to 5.52 (2021) showed a structurally disturbed Banki watershed and suffered massive anthropogenic transformation for livelihood. The Lu decreased by 70.33% from 1977 to 2021, where every stream order was either highly affected or disappeared in response to LULC change. Further, a decrease in the Rho coefficient (ρ)^20^ from 1977 to 2021 indicates shallowing storage capacity of the Banki watershed. Such an Anthropocene^46^ alteration in a small river reveals a disproportionately irreversible impact on the linear aspects of the subsequent high-order tributaries, e.g., the North Koel and Son in the Ganga River basin.

**Table 2:** Summary of linear aspects and drainage texture analysis of Banki watershed and subwatersheds.

Stream order (U)		No. of streams (Nu)		Bifurcation ratio (Rb)		Mean bifurcation ratio (Rbm)		Total length of streams (Lu) (km)		Mean length of streams (Lsm) (km)		Length ratio (RL) (Lur)		Rho coefficient (ρ)	
1977	2021	1977	2021	1977	2021	1977	2021	1977	2021	1977	2021	1977	2021	1977	2021
1	1	1133	128	4.05	3.46			715.64	143.68	0.63	1.12	2.58	1.02	0.64	0.29
2	2	280	37	3.94	4.11	4.14	5.52	277.76	140.87	0.99	3.81	1.89	2.43	0.48	0.59
3	3	71	9	3.23	9.00			146.71	57.92	2.07	6.44	1.68	1.47	0.52	0.16
4	4	22	1	5.50				87.30	39.48	3.97	39.48	4.36		0.79	
5		4		4				20.04		5.01		0.51		0.13	
6		1						39.48		39.48					
Total		1511	175					1286.92	381.95	52.15	50.85				

Summary of drainage texture analysis															
Watershed and subwatersheds	Drainage density (Dd)		Stream frequency (F_s_)		Drainage texture (Dt)		Constant of channel maintenance (C)		Infiltration number (I_f_)						
	1977	2021	1977	2021	1977	2021	1977	2021	1977	2021					
Banki River	2.48	0.74	2.92	0.34	12.26	1.42	0.40	1.36	7.24	0.25					
Saphi Nala	2.60	1.20	2.67	0.40	1.80	0.27	0.38	0.83	1.02	0.48					
Kajri Nala	2.43	0.82	2.69	0.26	1.97	0.19	0.41	1.22	1.11	0.22					
Longa Nala	2.86	0.73	3.91	0.29	3.53	0.26	0.35	1.37	1.37	0.21					
Sukhra Nadi	2.48	0.52	2.96	0.17	5.57	0.32	0.40	1.92	1.19	0.09					
Sugwa Nala	2.88	1.03	3.65	0.57	3.12	0.48	0.35	0.97	1.27	0.58					
Bailgarhwa Nala	2.67	0.99	2.88	0.44	2.36	0.36	0.37	1.01	1.08	0.43					
Hurhi Nala	3.04	0.00	3.20	0.00	1.81	0.00	0.33	0.00	1.05	0.00					
Koindi Nala	2.20	0.45	2.33	0.15	2.38	0.16	0.45	2.22	1.06	0.07					
Bhojpur Nala	2.13	0.00	1.97	0.00	1.00	0.00	0.47	0.00	0.92	0.00					
Baghi Nala	2.10	1.09	1.63	0.34	1.33	0.28	0.48	0.91	0.78	0.37					
Deogurwa Nala	3.18	0.00	3.96	0.00	2.14	0.00	0.31	0.00	1.24	0.00					
Beldahi Nala	3.14	1.23	4.14	0.60	3.66	0.53	0.32	0.81	1.32	0.49					
Satbahini Nala	2.99	0.71	3.52	0.53	3.18	0.48	0.33	1.41	1.18	0.75					
Sukh Nadi	3.36	1.07	4.39	0.60	2.42	0.33	0.30	0.93	1.31	0.56					
Sarsatia Nadi	2.27	0.56	2.69	0.23	3.41	0.29	0.44	1.78	1.18	0.41					
Banki Nala	2.19	0.83	2.53	0.45	2.42	0.43	0.46	1.21	1.16	0.54

The summary of the drainage texture analysis is given in Table 2. The Banki watershed showed a significant decrease in the drainage density (Dd)^20^, indicating spacing between streams of different orders increased from 1977 to 2021 due to the diversion of undulating hilly pediplain into agricultural land and increased the subsoil permeability with low surface runoff to the main trunk. The stream frequency (Fs)^20^ decreased from 2.92 (1977) to 0.34 (2021), indicating the alteration of a well-developed mature dendritic basin into the secluded 4th order Banki River, consequently cut off from existing geological formations and forests. The coarse drainage texture^20^ (Dt = 1.42), high constant of channel maintenance^22^ (C = 1.36), and low infiltration number^23^ (I_f_ = 0.25), thus indicating the porous surface, high infiltration capacity, and low runoff conditions, respectively, causing reduced river flow.

The drainage source map deals with the origin of first-order streams^20^, which decreased from 14 (1977) to 4 per square kilometer (2021) (Supplementary Fig. S4). The drainage frequency map deals with the number of streams per unit area^20^, which declined from 0 to 14 per km^2^ (1977) to 0 to 8 per km^2^ (2021), and dominant drainage frequency varied from 0 to 1.6 per km^2^ (Supplementary Fig. S5). The sixteen sub-watersheds have lost their 80–90% dendritic drainage pattern, while three sub-watersheds have become extinct (Supplementary Fig. S6). These extreme morphometric changes resulted in the loss of drainage confluence from 0–9 (1977) to 0–6 per square kilometer (1921), with the dominant drainage confluence varying from 0 to 1.2 per km^2^ (Supplementary Fig. S7). Consequently, a progressive loss of channel connectivity with the main truck and shifting of moderate and good groundwater potential zones (GWPZ) towards the poor GWPZ is distinctively visible (Supplementary Fig. S8). This outcome illustrates how river-aquifer interaction is significantly impacted by the transformation of the 6th order Banki River into the 4th order stream.

### Delineation of AB

**Figure 2:**
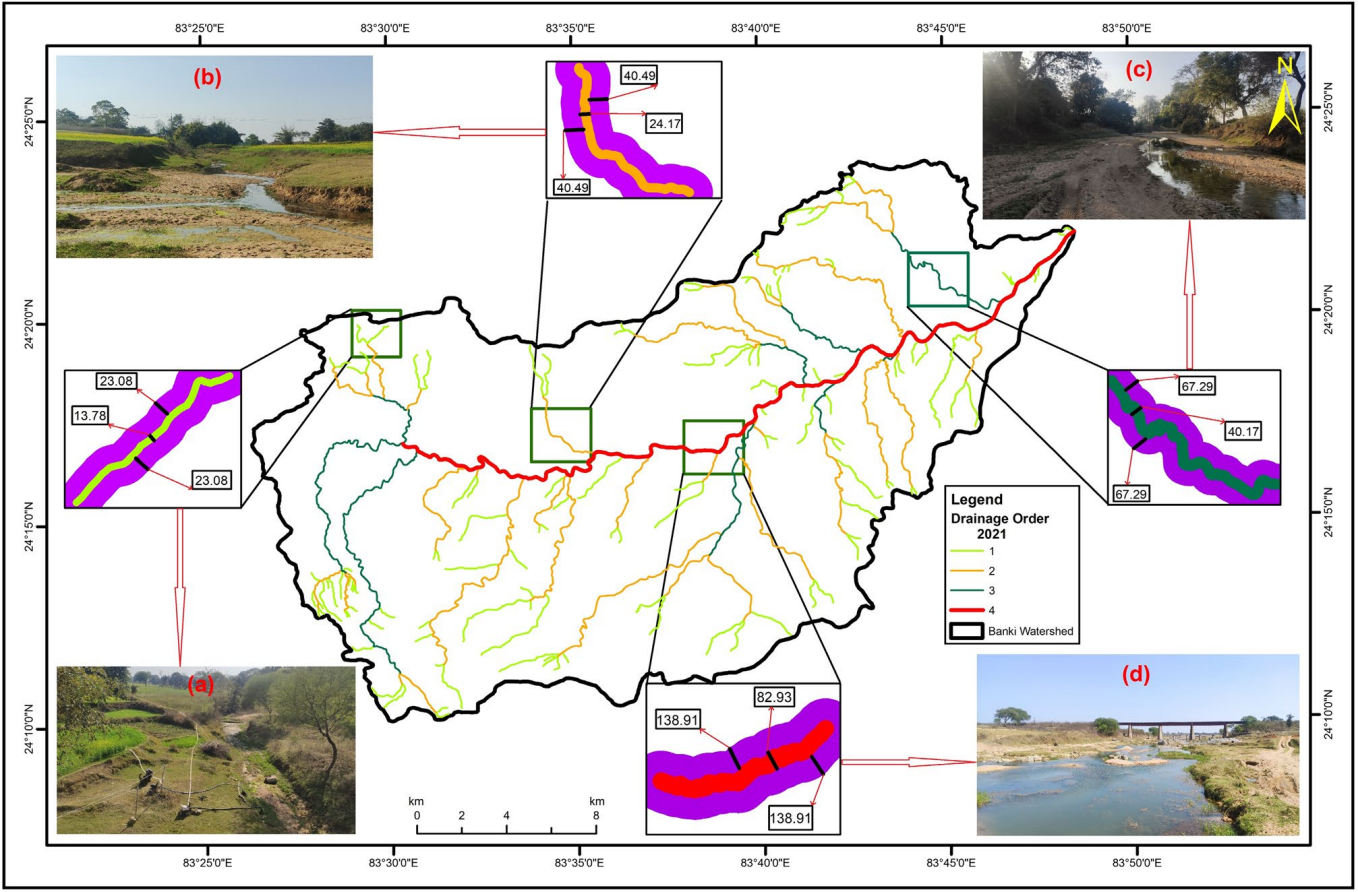
Delineation of W_RAB_ and W_LAB_ using watershed factor DDR and M_SW_. DDR is the ratio of Dd_1977_ and Dd_2021_. M_SW_ is the mean stream width. The W_RAB_ and W_LAB_ are half of the W_AB_ and greater than the M_SW_. (**a**) Displaying 1st order M_SW_, W_RAB,_ and W_LAB_. (**b**) Displaying 2nd order M_SW_, W_RAB,_ and W_LAB_. (**c**) Displaying 3rd order M_SW_, W_RAB,_ and W_LAB_. (**d**) Displaying 4th order M_SW_, W_RAB,_ and W_LAB_.

The delineation of administrative boundary (AB) is essential to protect existing river morphometry and restore the connectivity of stream orders recorded in 2021 with stream orders extracted from the SOI toposheets (1977). The results of the W_AB_ analysis are given in Table 3. The W_RAB_ and W_LAB_ are half of the W_AB_ and greater than the M_SW_, which is essential for AB fixation (Fig. 2). Among all primary morphometric parameters, Dd and DdR are the most robust, simple, and empirical to develop the W_AB_ in and around any stream order, irrespective of its width and length. It can be easily determined for each small tributary of the large rivers. Unlike the concept of the minimum and maximum river corridors to protect the natural boundary of the Ammer River^12^, the W_AB_ explains the drainage density-dependent fixed boundary of not only the 4th order Banki River but also delineates the W_AB_ along the 3rd, 2nd, and 1st order streams. Comparing protected river systems (PRSs) reveals the ecological significance of the drainage density at provincial and state scales^42^. We can learn lessons from the notified administrative boundaries of the protected areas^15^ and man-made infrastructures^47^, which are fixed in nature to maintain the present status and forecast future planning. Similarly, the W_AB_ is essential to protect the current ecological status of every stream and could be used in channelizing stormwater that does not reach the 4th order Bank River and restoring riverfronts.

**Table 3:** Summary of AB delineation.

Order	No. of streams	M_SW__(m)_	Dd_1977_ (km/km^2^)	Dd_2021_ (km/km^2^)	DdR = Dd_1977_/Dd_2021_	W_AB_ = M_SW_ × DdR (m)	W_RAB_ = W_AB_/2 (m)	W_LAB_ = W_AB_/2 (m)
1st order	128	13.78	2.48	0.74	3.35	46.16	23.08	23.08
2nd order	37	24.17				80.97	40.49	40.49
3rd order	9	40.17				134.57	67.29	67.29
4th order	1	82.93				277.83	138.91	138.91

### Threatened categories and criteria

The second most important question is how morphometric parameters can help classify the rivers into appropriately threatened categories. The percent change in the three most essential morphometric parameters (Nu, Lu, and Dd) is considered for ranking the Banki watershed into an appropriate threatened category (Table 4). The mean decrease in Nu, Lu, and Dd is > 70%, which classifies the Banki watershed into the “Critically Endangered” category. Before this study, the Banki watershed was not evaluated, data deficient, and least concerned. After detailed inventory, this watershed is adequately evaluated, data-rich, and highly concerned and depicts “Critically Endangered” status in the Ganga River basin. If we carry out this exercise for all such tributaries, it is possible to figure out the actual threatened status of the Ganga River basin.

**Table 4:** Summary of morphometric parameters evaluated for classifying the Banki watershed under the proposed RRLCC.

Morphometric parameter (s)	% decrease from the reference year (1977)							Mean decrease (%)	Conservation category	
	1st order	2nd order	3rd order	4th order	5th order	6th order	Overall			
Number of streams (Nu)	88.7	86.8	87.3	95.5	100.0	100.0	88.4	77.6	Critically endangered	
Total length of streams (Lu) (km)	79.9	49.3	60.5	54.8	100.0	100.0	70.3			
Drainage density (Dd) (km/km^2^)	80	49	61	55	100.0	100.0	74.1			

Ecological entity		Threatened category								
		Not evaluated (NE)	Data deficient (DD)	Least concern (LC)	Near threatened (NT)	Vulnerable (VU)	Endangered (EN)	Critically endangered (CR)	Near to extinction (NEX)	Extinct (EX)
Banki watershed	Before assessment (1977)	Yes	Yes	Yes	–	–	–	–	–	–
	After assessment (2021)	No	No	No	–	–	–	Yes	–	–

Threatened categories	Criteria									
Extinct (EX)	1. Entire loss of watershed, i.e., no Nu-Lu-Dd recorded									
Near to extinction (NEX)	1. Only Lu of the main trunk is recorded without Nu and Dd 2. Stream order can not be delineated 3. Extinction of subwatershed(s)									
Critically endangered (CR)	1. Average decrease in Nu-Lu-Dd is > 70% 2. Change in the stream order from 3 to 1, 4 to 2, 5 to 3, 6 to 4, 7 to 5 and so on in the main watershed or subwatershed(s) 3. Extinction of subwatershed(s)									
Endangered (EN)	1. Average decrease in Nu-Lu-Dd is < 70% to > 50% 2. Change in the stream order from 3 to 1, 4 to 2, 5 to 3, 6 to 4, 7 to 5 and so on in the main watershed or subwatershed(s) 3. Extinction of subwatershed(s)									
Vulnerable (VU)	1. Average decrease in Nu-Lu-Dd is < 50% to > 30% 2. Change in the stream order from 3 to 1, 4 to 2, 5 to 3, 6 to 4, 7 to 5 and so on in the main watershed or subwatershed(s) 3. No extinction of subwatershed(s)									
Near threatened (NT)	1. Average decrease in Nu-Lu-Dd is < 30% 2. No change in the stream order 3. No extinction of subwatershed(s)									

To expand the concept of threatened categories, we can learn lessons from the success of the IUCN Red List^48^. Similarly, the proposed “River Red List Categories and Criteria (RRLCC)” are given in Table 4. Seven threatened categories with corresponding criteria illustrate the extinction risk of river morphometry. Implementing such a scheme is essential to protect hydropattern (flow regime and hydroperiod)^15^ in the Ganga River basin while giving thrust to the morphometric conservation of the large and small tributaries. The Banki watershed (51,835 ha) is the lifeline for 0.332 million people living in 197 villages under seven district sub-divisions (1710 ha) and cultivating 22,200 ha of agricultural land in one of the most backward districts in the Ganga River basin, i.e., Garhwa^43^. This study provides reference data for the IUCN Protected Area Management Category VI, which deals with the sustainable use of natural resources^15^. Moreover, the AB and RRLCC are strengthening integrated river basin management (IRBM)^49^ or integrated water resources management (IWRM)^10,50^ of inland water bodies and transboundary rivers at local, regional, and global scales. The delineation of AB and implementation of RRLCC thus wisely support the new definition of IUCN protected areas (PA) inclusive of fresh waters^15^.

## Conclusion

The AB and RRLCC derivations have empirical merits because both are developed from the same morphometric parameters without any assumptions and deviations. The digitization of the reference maps (SOI toposheet 1977) before and after field inventory (2021) is robust, cost-effective, less time-consuming, free from mathematical or statistical biases, and portrays the actual status of the Banki watershed. We conclude that stream order diversity in terms of Nu, Lu, and Dd is an essential morphometric parameter imparting resistance and resilience to the watershed from environmental perturbations. With the threatened carrying capacity embedded in the local, regional, and transboundary Ganga River basin, the delineation of AB around small watersheds and their evaluation according to the RRLCC highlights the most immediate concern. In a nutshell, the AB and RRLCC offer one more chance to interlink fragmented landscapes within and outside the protected areas before the extinction of small streams and tributaries of transboundary rivers in the twenty-first century.

## Supplementary Information

Supplementary Figures.

## Data Availability

All data generated or analyzed during this study are included in the manuscript and supplementary information.
