# Supplementary material for: Morphometric delineation of administrative boundaries and classification of threatened categories of small watersheds in transboundary rivers
Source: Sci Rep. 2023 Jan 30;13:1652. doi: 10.1038/s41598-023-28913-5 (PMC9886851; doi:10.1038/s41598-023-28913-5)
Supplement: Supplementary file 1 — Supplementary Figures. [file 41598_2023_28913_MOESM1_ESM.docx]

**Fig. S1.** Location map of the study area. The picture on the top left shows the map of India and the picture on the top right shows the State of Jharkhand where the research area is located. The picture on the bottom is the study area map of the Banki watershed with elevation and drainage lines. Map created in Arc GIS 10.2 (ESRI Co., USA; (<https://www.esri.com/software/arcgis/arcgis-for-desktop>). Boundaries of India and Jharkhand are made with free vector data downloaded from DIVA-GIS (<https://www.diva-gis.org/gdata>).

**a**

**b**

**Fig. S2.** Spatial distribution maps rainfall pattern in the Banki watershed. (a) Average annual rainfall (1961-1990) variation from 1034.90 mm to 1097.48 mm. (b) Average annual rainfall (1991-2020) variation from 907.14 mm to 1042.06 mm. The maps were prepared using 0.25˚×0.25˚ gridded datasets of IMD (<https://www.imdpune.gov.in/Clim_Pred_LRF_New/Grided_Data_Download.html>) with help of IDW^21^ interpolation using Arc GIS 10.2 (ESRI Co., USA; <https://www.esri.com/software/arcgis/arcgis-for-desktop>).

**a**

**b**

**Fig. S3.** Land use and land cover (LULC) change. (a) Landsat 5 TM satellite imagery (1991). (b) Landsat 8 OLI satellite imagery (2021). These imageries (fall in path 142; row 043) were downloaded from USGS (<https://earthexplorer.usgs.gov/>) and classified by the ISODATA^31^ algorithm in Arc GIS 10.2 (ESRI Co., USA; <https://www.esri.com/software/arcgis/arcgis-for-desktop>).

**a**

**b**

**Fig. S4.** Spatial distribution of drainage source in the Banki watershed**.** (a) 1977 drainage source map was generated with the help of five SOI toposheets and IDW interpolation based on the number of 1^st^ order streams in each 1 km × 1 km fishnet grid using Arc GIS 10.2. (b) 2021 drainage source map was generated after the ground truth verification of the reference map (1977) and the updated map was created by repeating the same GIS processes.

**a**

**b**

**Fig. S5.** Spatial distribution of drainage frequency in the Banki watershed. (a) 1977 drainage frequency map was generated with the help of five SOI toposheets and IDW interpolation based on the total number of streams in each 1 km × 1 km fishnet grid using Arc GIS 10.2. (b) 2021 drainage frequency map was generated after the ground truth verification of the reference map (1977) and the updated map was created by repeating the same GIS processes.

**Fig. S6.** Comparison of stream order distribution between 1977 and 2021 in sixteen subwatersheds. The microscale changes in the stream orders and extinction of three subwatersheds depict the “Critically Endangered” status of the Banki watershed.

**a**

**b**

**Fig. S7.** Spatial distribution of drainage confluence in the Banki watershed. (a) 1977 drainage confluence map was generated with the help of five SOI toposheets and IDW interpolation based on the total confluence streams in each 1 km × 1 km fishnet grid using Arc GIS 10.2. (b) 2021 drainage confluence map was generated after the ground truth verification of the reference map (1977) and the updated map was created by repeating the same GIS processes.

**a**

**b**

**Fig. S8.** Groundwater potential zone (GWPZ) maps. (a) 1991. (b) 2021. ANN in MATLAB^33^ R2020b (https://in.mathworks.com/downloads/) and weighted overlay^40,41^ of input variables (elevation, slope, geomorphology, geology, lithology, lineament density, drainage density, soil texture, rainfall, and LULC) in Arc GIS 10.2 (ESRI Co., USA; <https://www.esri.com/software/arcgis/arcgis-for-desktop>).
